# Effect of preparation methods of CeO_2_ on the properties and performance of Ni/CeO_2_ in CO_2_ reforming of CH_4_

**DOI:** 10.1038/s41598-022-09291-w

**Published:** 2022-03-29

**Authors:** Zenan Ni, Xavier Djitcheu, Xiaoxu Gao, Jian Wang, Huimin Liu, Qijian Zhang

**Affiliations:** School of Chemical and Environmental Engineering, Liaoning University of Technology, Jinzhou, 121001 China

**Keywords:** Catalyst synthesis, Catalytic mechanisms, Heterogeneous catalysis

## Abstract

CO_2_ reforming of CH_4_ (CRM) is not only beneficial to environmental protection, but also valuable for industrial application. Different CeO_2_ supports were prepared to investigate the matching between Ni and CeO_2_ over Ni/CeO_2_ and its effect on CRM. The physicochemical properties of Ni/CeO_2_-C (commercial CeO_2_), Ni/CeO_2_-H (hydrothermal method) as well as Ni/CeO_2_-P (precipitation method) were characterized by XRD, N_2_ adsorption at − 196 °C, TEM, SEM–EDS, H_2_-TPR, NH_3_-TPD and XPS. Ni^0^ with good dispersion and CeO_2_ with more oxygen vacancies were obtained on Ni/CeO_2_-H, proving the influence on Ni/CeO_2_ catalysts caused by the preparation methods of CeO_2_. The initial conversion of both CO_2_ and CH_4_ of Ni/CeO_2_-H was more than five times that of Ni/CeO_2_-P and Ni/CeO_2_-C. The better matching between Ni and CeO_2_ on Ni/CeO_2_-H was the reason for its best catalytic performance in comparison with the Ni/CeO_2_-C and Ni/CeO_2_-P samples.

## Introduction

With the technology breakthrough in exploiting shale gas and combustible ice, searching clean approaches to utilize the main component CH_4_ efficiently has received extensive attention^1,2^. CO_2_ reforming of CH_4_, generally short for CRM, could convert greenhouse gas CH_4_ and CO_2_ to syngas (CO + H_2_). CRM is of great practical significance due to the following advantages: (1) the n(H_2_)/n(CO) ratio of the produced syngas is about 1, which could be directly used for Fischer–Tropsch synthesis; (2) CO_2_ and CH_4_ are greenhouse gases, and the utilization of them could really improve the ecological environment; (3) CRM requires high heat input, which means CRM could be employed for energy storage and transmission medium^3,4^.

Noble metal catalysts, such as Rh-^5^, Ru-^6^, Pd-^7^ based catalysts, exhibited good catalytic activity and strong anti-coking capacity in CRM. However, they could not be applied on an industrial scale because of their limited resources. On the contrary, the cheap and abundant non-noble metal catalysts, especially Ni-based catalysts, which give catalytic activities comparable to that of noble metal-based catalysts, have been widely studied^8–10^. Unfortunately, Ni-based catalysts generally suffer from poor stability. On one hand, at the high operating temperature of endothermic CRM (700–850 °C), Ni particles aggregate easily, which may reduce the number of active sites of the catalysts and eventually weaken the catalytic capacity^11^. On the other hand, filamentous carbon and active carbon are produced via CH_4_ cracking and CO disproportionation reactions. The accumulation and growth of these carbon species gradually cover and embed the active Ni particles and ultimately result in deteriorated catalyst stability^12,13^. Therefore, designing efficient Ni-based catalysts with strong anti-aggregation and anti-coking competence is critical for improving their stability in CRM.

With the aim to improve the anti-agglomeration competence of Ni-based catalysts, several approaches have been adopted, including (1) adding a structure promoter to stabilize Ni particles and (2) enhancing the Ni-support interaction to prevent the movement of Ni particles. For example, the Ni particles on Ni/SBA-15 catalyst modified by 1 wt% Sn (served as the structure promoter) were smaller and not easy to aggregate than those on unmodified catalyst^14^; the strong interaction between Ni and Al_2_O_3_ over Ni/Al_2_O_3_ catalyst, evidenced by the formation of NiAl_2_O_4_, prevents the aggregation of Ni particles greatly^15,16^. As for improving the anti-coking competence of Ni-based catalysts, the following routes are proved promising. (1) Adopting Ni-based catalysts with small Ni particles, since it is well accepted that smaller Ni particles exhibit stronger anti-coking competence^17,18^; (2) Adjusting the basic properties of Ni-based catalysts to facilitate CO_2_ adsorption and accelerate reverse CO disproportionation reaction, which could help to eliminate the deposited coke by the adsorbed CO_2_^19,20^. (3) Utilizing a material with strong oxygen storage capacity as support, which could serve as an oxygen reservoir for the elimination of deposited coke via oxidation reaction^21,22^.

Based on the research progress made by predecessors, it could be speculated that Ni/CeO_2_ catalyst with a large specific surface might be good for CRM, owing to (1) the Ni particles on a catalyst with a large specific surface area are generally well dispersed, and the interaction between Ni particles and CeO_2_ might be strong; (2) CeO_2_ is of basic properties^23^, which could facilitate CO_2_ adsorption and help to eliminate the deposited coke; (3) the oxidation–reduction property of CeO_2_ renders it as a strong oxygen reservoir^24^, which is ready to react with the deposited coke. Several groups have synthesized Ni/CeO_2_ catalysts and investigated their performance in CRM. However, controversial conclusions have been obtained. For instance, Shao et al*.* synthesized Ni/CeO_2_ via microemulsion method and reported that the as-prepared catalyst had smaller Ni particles (6–13 nm, with an average of 11 nm) and exhibited high activity in CRM^25^. Yahi et al*.* compared the catalytic performance of Ni/CeO_2_ prepared by auto-combustion method, sol–gel method and microemulsion method. It is discovered that, Ni/CeO_2_ prepared by microemulsion method (the average size of Ni particles were 11 nm) did not show any catalytic activity^26^. Holgado et al*.* got Ni/CeO_2_ (the size of Ni particles was in the range of 12–18 nm) by combustion method, which recorded a high activity but poor stability^27^. Rosen et al*.* synthesized Ni/CeO_2_ solid solution via exsolution method, which showed active and stable performance in CRM at 800 °C (The size of Ni particles was not clearly stated)^28^. Rodriguez et al*.* utilized theoretical calculation to investigate Ni/CeO_2_ catalyst and reported that it was a highly active catalyst for CRM even at a temperature as low as 700 K, and the strong interaction between Ni and CeO_2_ plays crucial roles in cleaving the C–H bond in CH_4_^29^. Ganduglia-Pirovano et al*.* considered that Ce^3+^ sites and the interaction between Ni and CeO_2_ worked in concert to cleave the C–H bond^30^. Zhu et al*.* treated Ni and CeO_2_ by plasma to get clean Ni–CeO_2_ interface, which was regarded to be responsible for its high activity in CRM^31^. Therefore, the preparation method of CeO_2_ has a great influence on the activity of Ni/CeO_2_ in CRM. Many studies focus on the small size of Ni, however, sometimes, Ni/CeO_2_ catalysts with small size of Ni still exhibit unsatisfactory catalytic performance. The controversial conclusions might be caused by the poor matching between Ni and CeO_2_ supports, and it is difficult to get a generalized guidance for the rational design of efficient catalysts.

For studying the impact of the synthesis procedures of CeO_2_ supports on the catalytic performance of the binary oxides, the low Ni content and the introduction method of Ni were control the same, and CeO_2_ prepared by three different methods were used as the supports of Ni/CeO_2_ catalysts. The properties of three kinds of CeO_2_ and their supported Ni-based catalysts were characterized, and the performance of Ni/CeO_2_ catalysts in CRM were evaluated.

## Experimental section

### Catalyst preparation

Chemicals in this study were of analytical grade and used as received. CeO_2_-H was used to denote CeO_2_ prepared by hydrothermal method. It was prepared via the following procedure: 11.2 g CeCl_3_·7H_2_O and 9.4 g cetyltriethylammnonium bromide (CTAB) were firstly dissolved in 550 mL deionized water, to which 25 mL 25 wt% NH_3_ solution was then added drop-wisely. The slurry was stirred at room temperature for 1 h and then treated at 90 °C for 30 h with reflux. After cooling down to room temperature, it was washed by deionized water and acetone to neutral. Finally, it was dried at 80 °C for 24 h and calcined in air at 700 °C (at a heating ramp of 2 °C min^−1^) for 5 h to obtain CeO_2_-H. CeO_2_-P was used to denote CeO_2_ prepared by precipitation method. It was prepared via the following procedure: 250 mL 0.2 wt% NH_3_ solution and 250 mL 16.0 g L^−1^ CeCl_3_ solution were added drop-wisely to another 250 mL 0.2 wt% NH_3_ solution under vigorous stir. The slurry was stirred at room temperature for 1 h and then aged for 12 h. After centrifugation, it was washed by deionized water and acetone to neutral. Finally, it was dried at 80 °C for 24 h and calcined in air at 700 °C (at a heating ramp of 2 °C min^−1^) for 5 h to obtain CeO_2_-P.

CeO_2_-H, CeO_2_-P, together with commercial CeO_2_ (denoted as CeO_2_-C) were used as supports of Ni-based catalysts. Ni/CeO_2_ catalysts were prepared by loading a certain amount of Ni(NO_3_)_2_ onto the CeO_2_ supports using the wetness impregnation method. After impregnating for 10 h, the samples were evaporated, dried at 80 °C and calcined at 700 °C for 5 h. The as-prepared catalysts were denoted as Ni/CeO_2_-H, Ni/CeO_2_-P and Ni/CeO_2_-C, respectively.

### Catalyst characterization

The crystalline structures of CeO_2_ and Ni/CeO_2_ catalysts were determined by X-ray diffraction (XRD) method on an X-Pert diffractometer equipped with graphite monochromatized Cu-Kα radiation. The specific surface areas were determined using a surface area analyzer (BEL Sorp-II mini, BEL Japan Co., Japan) with the Brunauer–Emmett–Teller (BET) method. Shape of the samples were observed using a transmission electron microscope (TEM-16-TS-008) and scanning electron microscopy (SEM) equipped with energy-dispersive X-ray spectroscopy (EDS). H_2_-temperature programmed reduction (TPR), CO_2_-temperature programmed desorption (CO_2_-TPD) and NH_3_-temperature programmed desorption (NH_3_-TPD) profiles were carried out over Quantachrome instrument, with the temperature raising from room temperature to 900 °C at a heating rate of 10 °C min^−1^. X-ray photoelectron spectroscopy (XPS) was carried out on Thermo Fisher equipped with Al Kα radiation. Binding energies were calibrated by carbon (C 1*s*, 284.6 eV). The amount of coke deposited on the spent catalysts was characterized by Thermogravimetry (TG). The composition of the samples was determined by inductively coupled plasma-optical emission spectrometry (ICP-OES) on a 730 Series ICP-OES by Agilent Technologies.

### Catalyst evaluation

CRM reaction was conducted in a fixed-bed reactor under atmospheric pressure. A portion of 0.20 g catalyst was packed uniformly in the temperature-constant zone of a quartz tube. Before reaction, the catalysts were reduced by 20% H_2_/Ar at 700 °C for 1 h. Then 20.0 mL min^−1^ (STP) CH_4_ and 20.0 mL min^−1^ (STP) CO_2_ were introduced into the reactor as reactants, with WHSV of 4.3 h^−1^ for CH_4_ and 11.8 h^−1^ for CO_2_. After removing the byproduct water via an ice trap, the effluent gas was analyzed by a gas chromatography equipped with a TDX-01 column to determine the relative amounts of CH_4_, CO, CO_2_ and H_2_, and the flow rate of the effluent gas was measured with a flow meter.

## Results and discussion

### Crystalline structure of CeO_2_ and Ni/CeO_2_ catalysts

**Figure 1:**
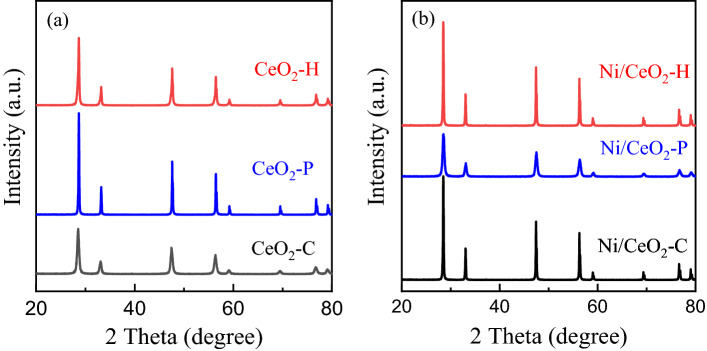
XRD patterns of (**a**) CeO_2_ supports and (**b**) Ni/CeO_2_ catalysts.

The crystalline structures of CeO_2_ and Ni/CeO_2_ catalysts were characterized by XRD, and the results were displayed in Fig. 1. The intense and sharp diffraction peaks at 28.6, 33.1, 47.5, 56.3, 59.1, 69.4, 77.0 and 79.1 in Fig. 1a were assigned to fluorite-structured CeO_2_ (JSPDS 34-394)^32,33^, indicating CeO_2_-H and CeO_2_-P were successfully synthesized. The crystalline structure of CeO_2_ changed little after the loading of Ni (Fig. 1b), and no obvious diffraction peaks assigned to Ni species were observed. ICP results showed that the actual Ni loading for Ni/CeO_2_-H (0.80 wt%), Ni/CeO_2_-C (0.84 wt%) and Ni/CeO_2_-P (0.81 wt%) was low, which may below the detection limit of XRD analysis.

### Textural properties of CeO_2_ and Ni/CeO_2_ catalysts

**Figure 2:**
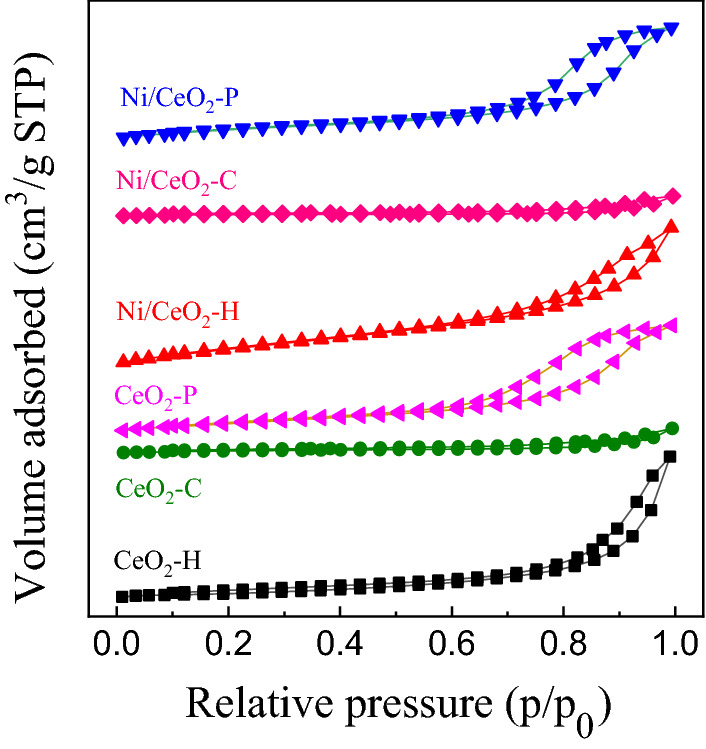
N_2_ adsorption–desorption isotherms of CeO_2_ based catalysts.

The textual properties of CeO_2_ and Ni/CeO_2_ catalysts were obtained by N_2_ adsorption–desorption technique, and N_2_ adsorption–desorption isotherms of different Ni/CeO_2_ based catalysts were shown in Fig. 2. The H4 hysteresis loops of Ni/CeO_2_-H and Ni/CeO_2_-P suggested the presence of mesoporous structures and the volume of mesoporous (V_mes_) (Table 1) verified it. The specific surface area (S_BET_) was also given in Table 1, and as can be seen that after the introduction of 0.8 wt% Ni on CeO_2_ supports, S_BET_ of all the three catalysts decreased slightly and V_mes_ remained the same.

**Table 1:** Textural properties of CeO_2_ based catalysts.

Sample	S_BET_ (m^2^ g^−1^)	V_mes_^a^ (cm^3^ g^−1^)	Crystallite size (nm)
CeO_2_-C	7.3	0.02	60–100
CeO_2_-P	39. 6	0.11	10–40
CeO_2_-H	25.7	0.13	10
Ni/CeO_2_-C	5.4	0.02	60–100
Ni/CeO_2_-P	35.5	0.11	10–40
Ni/CeO_2_-H	22.4	0.13	10

^a^V_mesopore_ (= V_total_ – V_micropore_), P/P_0_ = 0.99.

### Morphologies of Ni/CeO_2_ catalysts

**Figure 3:**
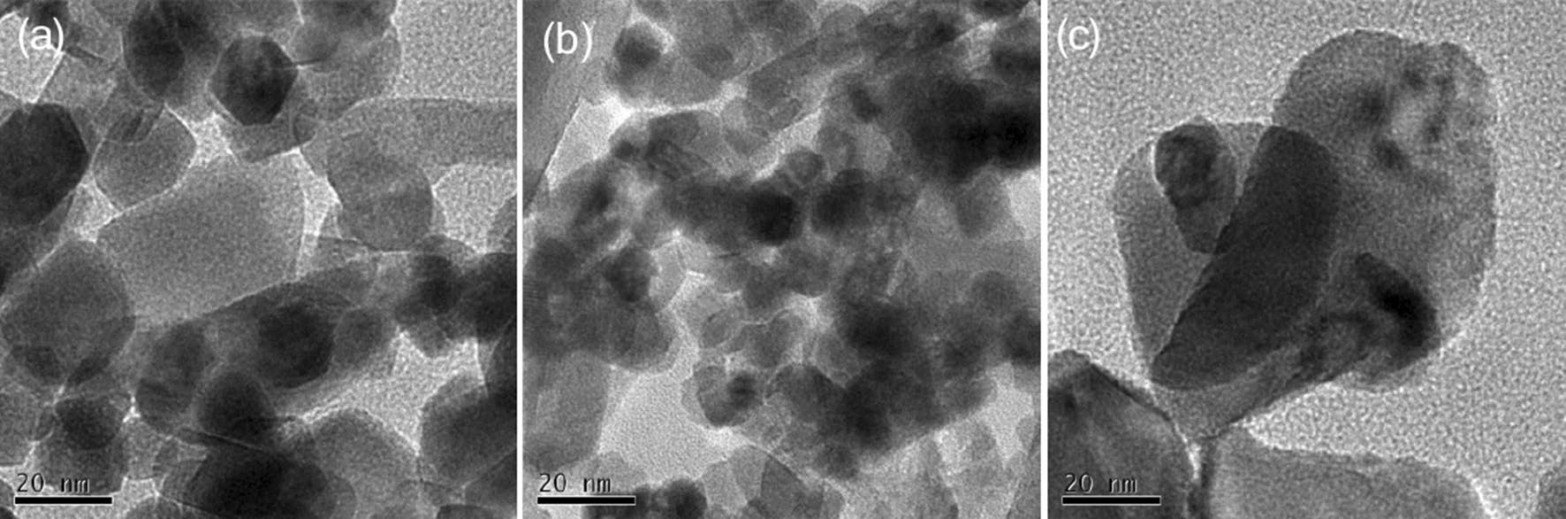
TEM images of (**a**) Ni/CeO_2_-P, (**b**) Ni/CeO_2_-H and (**c**) Ni/CeO_2_-C.

The morphologies of Ni/CeO_2_ catalysts were observed via TEM and SEM. Over the TEM images of three Ni/CeO_2_ catalysts (Fig. 3), nanoparticles assigned to CeO_2_ could be clearly observed. Obviously, CeO_2_ over Ni/CeO_2_-P was somewhat large, in the range of 10–40 nm (Fig. 3a); CeO_2_ over Ni/CeO_2_-H was relatively uniform and small, around 10 nm (Fig. 3b); Meanwhile, the size of CeO_2_ over Ni/CeO_2_-C was the largest, 60–100 nm (Fig. 3c). Notably, no Ni species were detected in the TEM images, inferring that Ni was well dispersed on three catalysts, and the crystallite size of CeO_2_ in the three catalysts barely changed before and after the introduction of Ni (Table 1), which may be related to the low Ni loading.

**Figure 4:**
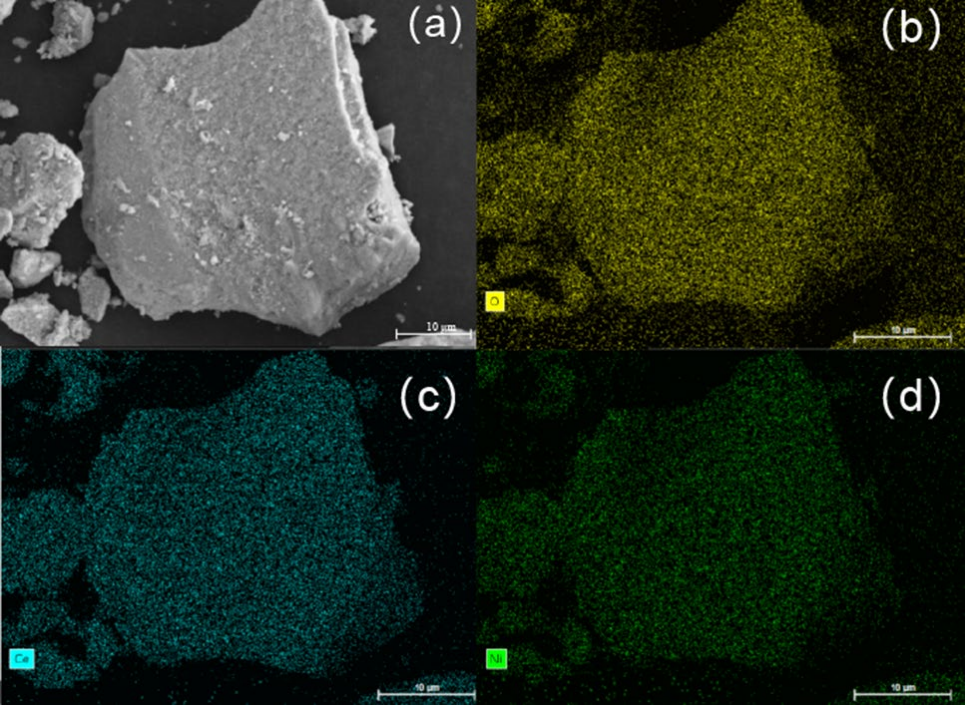
SEM image and the corresponding mapping of Ni/CeO_2_-H catalysts: (**a**) SEM image; (**b**) O element; (**c**) Ce element; (**d**) Ni element.

Furthermore, the chemical analysis of the mixed oxides by SEM and the corresponding EDS were conducted to investigate the elemental distribution and the homogeneity of the three Ni/CeO_2_ catalysts. As was shown in Fig. 4, Ni, O and Ce elements were uniformly distributed on Ni/CeO_2_-H catalyst. The same results could also be obtained for Ni/CeO_2_-P (Fig. S1) and Ni/CeO_2_-C (Fig. S2), which further proved that Ni has a good dispersion state on the three Ni/CeO_2_ catalysts.

### Acidic-basic properties of Ni/CeO_2_ catalysts

**Figure 5:**
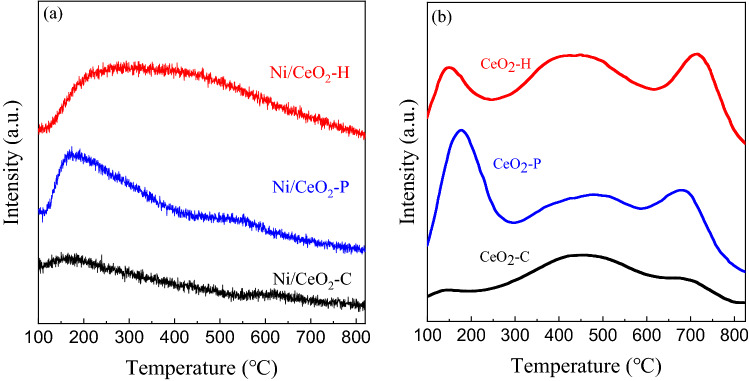
NH_3_-TPD profiles of different (**a**) Ni/CeO_2_ and (**b**) CeO_2_.

Acidic properties of different Ni/CeO_2_ catalysts were characterized by NH_3_-TPD, and the results were illustrated in Fig. 5. As was shown in Fig. 5a, there was only one obvious desorption peak (150 °C) over Ni/CeO_2_-C corresponding to the weak acid sites. More weak (187 °C) and strong (550 °C) acid sites, especially for weak, were observed on Ni/CeO_2_-P. It was noting that on Ni/CeO_2_-H, there was a big and wide peak ranging from 130 to 900 °C, corresponds to the maximum amount of weak, medium and strong acid sites. The acid properties of different CeO_2_ supports were also tested and shown in Fig. 5b. There were more weak acids on CeO_2_-P and more medium as well as strong acid sites on CeO_2_-H than CeO_2_-C. Such differences came from different preparation methods of CeO_2_.

**Figure 6:**
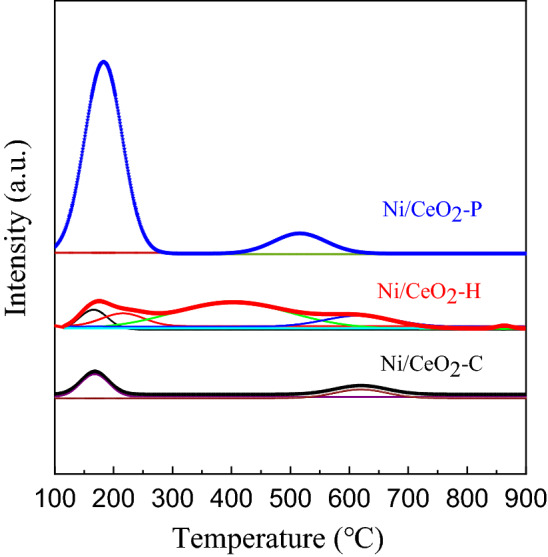
CO_2_-TPD profiles of Ni/CeO_2_-P, Ni/CeO_2_-H and Ni/CeO_2_-C.

CO_2_-TPD experiment was performed to study the basic properties of Ni/CeO_2_-P, Ni/CeO_2_-H and Ni/CeO_2_-C, and the results were shown in Fig. 6 and Table 2. It could be found that two main peaks of CO_2_ desorption were observed at 168 °C and 620 °C over Ni/CeO_2_-C and the overall desorption amount was the least, proving the weak and less basic sites of Ni/CeO_2_-C. There were two peaks centered at 183 °C and 517 °C and an incredible amount of CO_2_ desorption was detected on Ni/CeO_2_-P, indicating a large number of weak basic sites on Ni/CeO_2_-P. For Ni/CeO_2_-H, there were four peaks corresponding to weak (173 °C), medium weak (402 °C), medium strong (622 °C) and strong (863 °C) basic sites, showing the diverse basic properties of the sample prepared by hydrothermal method. CO_2_ adsorption values of prepared catalysts in Table 2 demonstrated it. It should be noted that CO_2_ was one of the reactants, and the highest desorption temperature (863 °C) seems to mean that the binding effect between Ni/CeO_2_-H catalyst and CO_2_ was strong, which may be conducive to the CO_2_ reaction.

**Table 2:** CO_2_ adsorption values for TPD experiments.

Sample	Peak (℃)	Density (μmolCO_2_ g^−1^)
Ni/CeO_2_-C	168	4.3
	620	2.8
Ni/CeO_2_-P	183	23.5
	517	5.6
Ni/CeO_2_-H	173	4.2
	402	6.6
	622	2.4
	863	0.1

### The state of Ni and CeO_2_ over Ni/CeO_2_ catalysts

**Figure 7:**
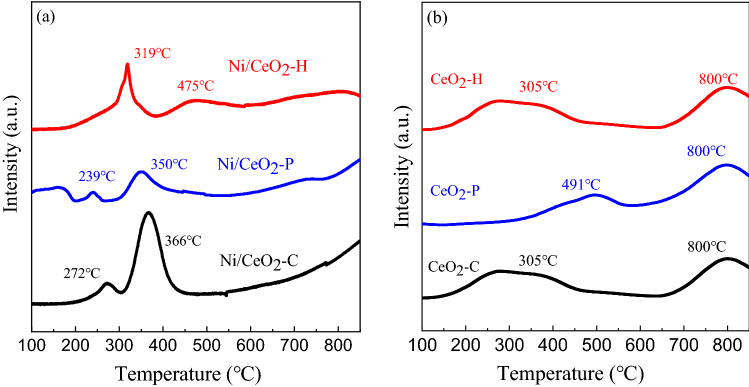
H_2_-TPR profiles of (**a**) Ni/CeO_2_ and (**b**) CeO_2_.

H_2_-TPR experiment was performed to study the state of Ni and CeO_2_ during the reducing atmosphere, and the results were shown in Fig. 7. It could be found in Fig. 7a that there were three major peaks at the temperature of 100–600 °C, 366 °C for Ni/CeO_2_-C, 350 °C for Ni/CeO_2_-P and 319 °C for Ni/CeO_2_-H, respectively. It is generally accepted that the smaller the NiO particle size, the lower the hydrogen consumption temperature. Therefore, it could be inferred that the particle size of bulk NiO particles decreased in the order of Ni/CeO_2_-C, Ni/CeO_2_-P and Ni/CeO_2_-H. There were also two relatively small peaks on Ni/CeO_2_-P (239 °C) and Ni/CeO_2_-C (272 °C), which were attributed to surface NiO. The H_2_-TPR profiles of three CeO_2_ were depicted in Fig. 7b, and as was shown that two obvious peaks could be observed on all samples: low-temperature peak for surface shell reduction (Ce^4+^ to Ce^3+^) and high-temperature peak for bulk reduction. Comparing the two figures in Fig. 7, the shoulder peak at 476 °C on Ni/CeO_2_-H should be attributed to surface CeO_2_.

### Surface structures of Ni/CeO_2_ catalysts

**Figure 8:**
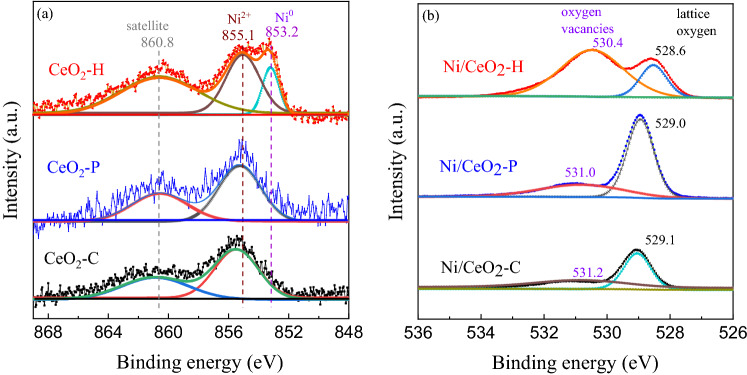
XPS spectra of (**a**) Ni 2*p*_3/2_ and (**b**) O 1*s* in the reduced Ni/CeO_2_.

XPS was used to clarify the surface chemical environment and the valence state of elements on the reduced Ni/CeO_2_ catalysts, and the results were shown in Fig. 8. As shown in Fig. 8a, there were three peaks located at about 853.2, 855.1, and 860.8 eV, attributed to Ni^0^, Ni^2+^, and the satellite peak of Ni 2*p*_3/2_ on reduced Ni/CeO_2_-H^34^. In contrast, Ni^0^ was almost absent on Ni/CeO_2_-C and Ni/CeO_2_-P. Figure 8b showed the XPS spectra of O 1*s* region for different Ni/CeO_2_ catalysts. O 1*s* peaks at 528.6–529.1 eV were assigned to lattice oxygen, while peaks at 530.4–531.2 eV were assigned to oxygen vacancies. In conclusion, after reduction treatment, the most Ni^0^ and oxygen vacancies were obtained over Ni/CeO_2_-H among the three catalysts.

### Catalytic performance of Ni/CeO_2_ catalysts in CRM

**Figure 9:**
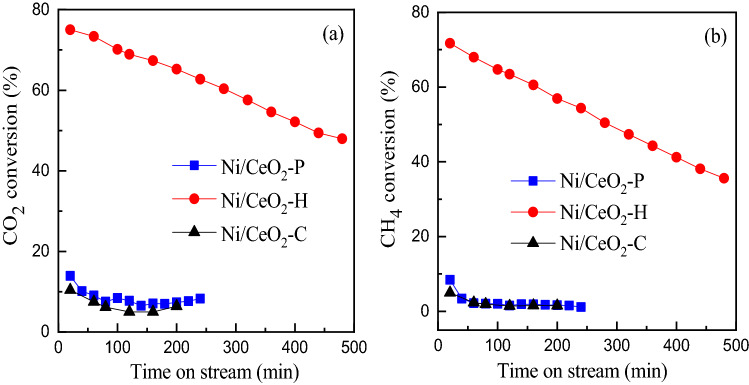
(**a**) CO_2_ conversion and (**b**) CH_4_ conversion of prepared Ni/CeO_2_ catalysts as a function of time on stream. Reaction conditions: 0.20 g catalyst, CH_4_ 20.0 mL min^−1^ (STP), CO_2_ 20.0 mL min^−1^ (STP), 700 °C.

The catalytic performance of Ni/CeO_2_ catalysts in CRM were evaluated in a fixed bed reactor and the results were shown in Fig. 9. It was clear that Ni/CeO_2_-H exhibited high catalytic activity (The initial CO_2_ and CH_4_ conversions were 75% and 71%, respectively). On the contrary, the catalytic performance of Ni/CeO_2_-P and Ni/CeO_2_-C was poor (the initial CO_2_ and CH_4_ conversions were within 20%). The conversion of more than five times proved the superiority of Ni/CeO_2_-H. Due to the reverse water gas shift reaction, CO_2_ conversion was higher than CH_4_ conversion over all the three Ni/CeO_2_ catalysts. After 480 min time on stream, CO_2_ conversion dropped from 75 to 48% and CH_4_ conversion dropped from 71 to 35%. The unsatisfied stability of Ni–CeO_2_-H might be caused by coke. For comparison, CRM reaction of CeO_2_-H, CeO_2_-P and CeO_2_-C was also evaluated. The activity of three prepared bare CeO_2_ supports in this work was nearly inert (both CH_4_ and CO_2_ conversion were less than 3%), which indicated that Ni played a crucial role in CRM reaction, and the preparation method of CeO_2_ has a great influence on the matching between Ni and CeO_2_.

### Crystalline structure of spent Ni/CeO_2_ catalysts

**Figure 10:**
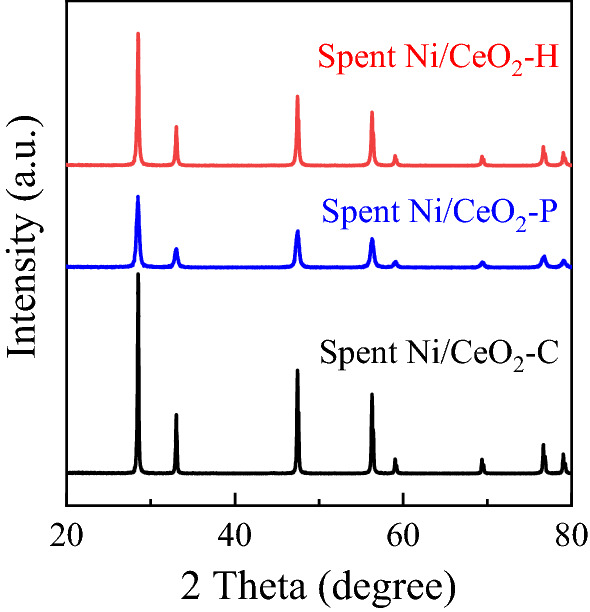
XRD patterns of spent Ni/CeO_2_ catalysts.

The crystalline structure of spent Ni/CeO_2_ catalysts was further characterized by XRD, and the results were displayed in Fig. 10. The nearly unchanged diffraction peaks between fresh and spent Ni/CeO_2_ catalysts revealed that the crystalline structure of CeO_2_ did not change during the reaction atmosphere. No obvious diffraction peaks assigned to Ni species were observed.

### TG of spent Ni/CeO_2_ catalysts

**Figure 11:**
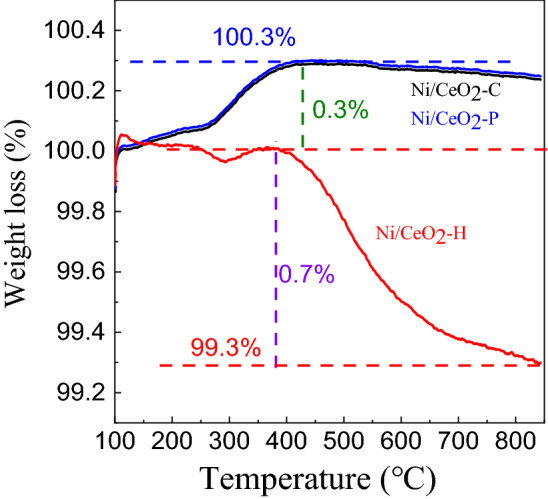
TG curves of the spent Ni/CeO_2_-C, Ni/CeO_2_-P and Ni/CeO_2_-H. Reaction conditions: 0.20 g catalyst, CH_4_ 20.0 mL min^−1^ (STP), CO_2_ 20.0 mL min^−1^ (STP), 700 °C, 480 min.

The coking behavior of spent catalysts of Ni/CeO_2_-C, Ni/CeO_2_-P and Ni/CeO_2_-H was tested by TG, and the results were shown in Fig. 11. As can be seen, the similar two stages of weight change could be found over Ni/CeO_2_-C and Ni/CeO_2_-P, and the 0.3% increase in mass should be attributed to the oxidation process of Ni and the oxygen species adsorbed by oxygen vacancies of CeO_2_ supports in the first stage (100–425 °C). Since the catalytic activity was weak, there was almost no weight loss for Ni/CeO_2_-C and Ni/CeO_2_-P in the second stage (425–850 °C). As for Ni/CeO_2_-H, almost no weight change was observed during the first parts (100–382 °C), which may possibly because Ni and oxygen vacancies with catalytic activity were occupied by coke, and the 0.7% weight loss in the second stage (380–850 °C) should be corresponded to the amount of coke. The BET surface area of the spent Ni/CeO_2_-H (17.9 m^2^ g^−1^) proved the covering effect of coke on the catalyst.

### Matching of Ni and CeO_2_ on Ni/CeO_2_ catalysts in CRM

The activation of Ni to CH_4_ and CeO_2_ to CO_2_ are very important for CRM reaction. On one hand, small Ni particles have a strong ability to activate the C-H bond of alkanes, while the aggregated Ni could easily induce CH_4_ cracking to produce coke^12^. Therefore, it is very important to prepare small Ni particles with high dispersion. On the other hand, basic CeO_2_ with more oxygen vacancies is beneficial to the chemisorption and dissociation of CO_2_^35^. The results of H_2_-TPR (Fig. 7) and XPS (Fig. 8) manifested that small Ni and CeO_2_ with more oxygen vacancies were obtained on Ni/CeO_2_-H through hydrothermal method, and the matching of Ni and CeO_2_ has been effectively demonstrated by the catalytic performance (Fig. 9). What was more, CO_2_ dissociation and thereafter oxidation of carbon deposit can take place over low acidic catalyst system. So, Low acid catalyst system is expected to improve the stability of catalysts to a certain extent.

## Conclusions

Three Ni/CeO_2_ catalysts were prepared by different method, and the matching between Ni and different CeO_2_ supports as well as their effects on CRM reaction have been well studied. The conversions of CO_2_ and CH_4_ of Ni/CeO_2_-P were slightly better than that of Ni/CeO_2_-C, and the catalytic activity of Ni/CeO_2_-H was more than 5 times that of Ni/CeO_2_-P or Ni/CeO_2_-C. According to the results of related characterization and evaluation, it can be concluded that the better matching of Ni and CeO_2_, including Ni^0^ with good dispersion and CeO_2_ with more oxygen vacancies, was the fundamental reason for improving the reaction activity of CRM. It is a remarkable fact that coke has a great influence on the stability of catalysts, and necessary experiments are still needed. This work investigated and demonstrated the advantages and differences of hydrothermal preparation of CeO_2_ supports and throw new light on the design of highly efficient Ni/CeO_2_ catalysts for CRM.

## Supplementary Information

Supplementary Figures.
