# Supplementary material for: Effect of preparation methods of CeO2 on the properties and performance of Ni/CeO2 in CO2 reforming of CH4
Source: Sci Rep. 2022 Mar 29;12:5344. doi: 10.1038/s41598-022-09291-w (PMC8964754; doi:10.1038/s41598-022-09291-w)
Supplement: Supplementary file 1 — Supplementary Figures. [file 41598_2022_9291_MOESM1_ESM.docx]

SUPPORTING INFORMATION

**Effect of preparation methods of CeO_2_ on the properties and performance of Ni/CeO_2_ in** **CO_2_ reforming of CH_4_**

*Zenan Ni*, Xavier Djitcheu, Xiaoxu Gao, Jian Wang, Huimin Liu*, Qijian Zhang*

School of Chemical and Environmental Engineering, Liaoning University of Technology, Jinzhou 121001, China

Corresponding Author:

Zenan Ni, email: hgnzn@lnut.edu.cn

Huimin Liu, email: liuhuimin08@tsinghua.org.cn

Fig. S1. SEM image and the corresponding mapping of Ni/CeO_2_-P catalysts: (a) SEM image; (b) O element; (c) element; (d) Ni element.

Fig. S2. SEM image and the corresponding mapping of Ni/CeO_2_-C catalysts: (a) SEM image; (b) O element; (c) element; (d) Ni element.
